# Alcohol-Related Pancreatic Damage: Mechanisms and Treatment

**Published:** 1997

**Authors:** Minoti V. Apte, Jeremy S. Wilson, Mark A. Korsten

**Affiliations:** Minoti V. Apte, M.D., M. Med. Sci., is a research officer and Jeremy S. Wilson, M.D., Ph.D., is an associate professor at the Gastrointestinal Unit, Pancreatic Research Group, Prince of Wales Hospital, Randwick, Australia. Mark A. Korsten, M.D., is director of gastrointestinal endoscopy at the Alcohol Research Center, Mount Sinai School of Medicine, New York, New York

**Keywords:** alcoholic pancreatitis, prevalence, pathogenesis, diagnosis, treatment

## Abstract

Pancreatitis is a potentially fatal inflammation of the pancreas often associated with long-term alcohol consumption. Symptoms may result from blockage of small pancreatic ducts as well as from destruction of pancreatic tissue by digestive enzymes. In addition, by-products of alcohol metabolism within the pancreas may damage cell membranes. Research on the causes of pancreatitis may support more effective disease management and provide hope for a potential cure.

An association between alcohol abuse and pancreatic injury was reported as early as 1878 (Friedreich 1878). Alcoholic pancreatitis is a potentially fatal illness that may be short term (i.e., acute) or long term (i.e., chronic). The relationship between acute and chronic pancreatitis is complex. Symptoms shared by acute and chronic pancreatitis include disabling abdominal pain and interference with normal pancreatic functions. Although the prevalence of alcoholic pancreatitis in the population is unknown, clinicians usually agree that both acute and chronic alcoholic pancreatitis are responsible for a significant amount of illness and death in the United States. This article discusses the extent of the problem, clinical aspects, diagnosis, development (i.e., pathogenesis), and treatment of both acute and chronic alcohol-related pancreatitis.

## The Healthy Pancreas

**Figure 1:**
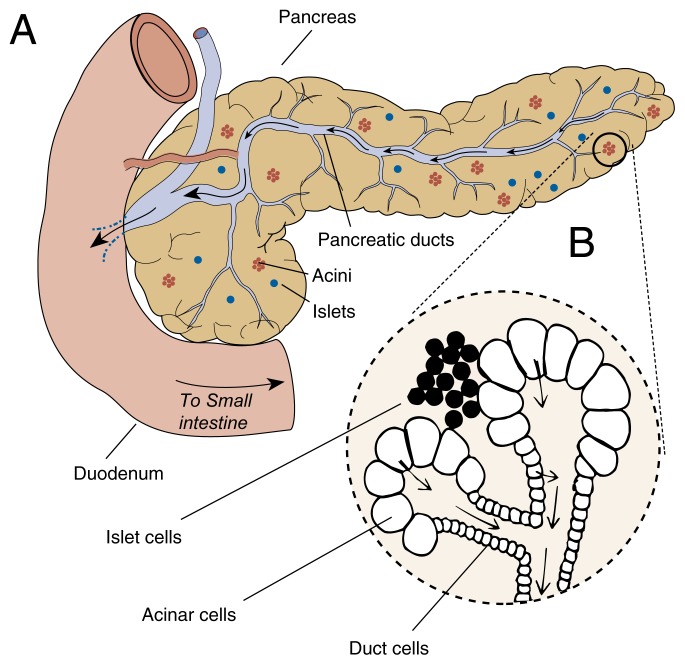
The human pancreas. (A) View of the pancreas showing clusters of acinar cells (i.e., acini), islet cells (i.e., islets), and pancreatic ducts. (B) An enlargement of a secretory region of the pancreas. Acini secrete digestive enzymes into the small intestine, islets secrete the hormones insulin and glucagon into the bloodstream to regulate blood glucose concentration, and duct cells secrete bicarbonate to regulate small intestine acidity.

The pancreas lies deep within the abdomen, behind the stomach. The pancreas serves two major functions (figure 1). First, certain cells (i.e., islet cells) dispersed throughout the pancreas play the role of an endocrine gland by producing two crucial hormones that regulate blood-sugar (i.e., glucose) levels: insulin and glucagon. Poorly regulated blood glucose can produce symptoms associated with diabetes. The hormones produced by these cells are released directly into the bloodstream. Second, another specialized group of cells (i.e., acinar cells) secrete digestive enzymes into the small intestine through tubes (i.e., ducts). In support of its digestive function, the pancreas also secretes bicarbonate through these same ducts. Pancreatic bicarbonate, a chemical similar to household baking soda, helps adjust and maintain the relatively weak acidity required for the action of intestinal digestive enzymes.

Pancreatitis arises in the acinar cells. However, inflammatory damage can destroy all parts of the pancreas—the islet cells as well as the acinar cells. Any disorder that affects the digestion of food or the subsequent metabolism of digested food in the bloodstream is likely to have serious consequences for the entire body.

## The Extent of the Problem

Since Friedreich’s initial observation, many studies have confirmed that excessive alcohol intake is associated with pancreatic damage. However, the proportion of cases of pancreatitis attributed to alcohol varies widely among countries and even among different studies in the same country. In the United States, for instance, the reported incidence of pancreatitis attributed to alcohol ranges from 5 to 90 percent. This huge variation may be related to the difficulties in accurately identifying alcohol abuse and to differences in the populations studied. For example, in a Veterans Affairs hospital (where the prevalence of alcoholism among patients is high) (Steinberg and Tenner 1994), the number of cases of alcohol-related pancreatitis is likely to be higher than that in a rural community hospital.

The mortality rate of patients with alcoholic pancreatitis is about 36 percent higher than that of the general population. Approximately 50 percent of patients with alcoholic pancreatitis die within 20 years of onset of the disease. Only 20 percent of deaths occurring before a patient’s life expectancy are attributed to pancreatitis or its complications; most of these deaths are attributed to the effects of alcohol or smoking on other organs such as the liver.

The increased risk of pancreatic cancer reported in heavy alcohol users (i.e., people who consume 10 to 12 standard drinks per day)^1^ by earlier studies has not been confirmed by more recent investigations. One complicating factor in some of the studies is cigarette smoking, which is commonly associated with alcohol abuse. When the effect of cigarette smoking was controlled for statistically, no association was found between alcohol abuse and pancreatic cancer. It has been suggested that the specific risk of pancreatic cancer among alcoholics may be limited to those alcoholics who develop chronic pancreatitis (Ahlgren 1996). This notion is not unreasonable, given that an excess relative risk of pancreatic cancer has been found in nonalcoholic types of pancreatitis, including a hereditary form of the disease (Andren-Sandberg and Lowenfels 1997). The relationship of alcohol-related pancreatitis to pancreatic cancer, however, is less clear, again because of possible contributing factors, such as malnutrition and smoking.

## Medical Aspects

Alcoholic pancreatitis usually occurs in men in their forties. Initial symptoms include vomiting as well as acute abdominal pain, which may be localized to the back and upper abdomen and is relieved by leaning forward. In mild cases, the pain may last 2 to 3 days; the short-term prognosis in such cases is very good. In severe cases, however, the pain may persist for several weeks and the risk of death rises to about 30 percent. Less commonly, pancreatitis can be completely painless and is only diagnosed from symptoms of insufficient pancreatic function, such as diabetes and steatorrhea (excess fat in feces). One complication of pancreatitis is localized masses of dead tissue and old blood walled off between the pancreas and surrounding organs (i.e., pseudocysts). If a pseudocyst becomes infected, it can invade the pancreas and become an abscess.

Approximately 5 to 6 years after the onset of the disease (especially in patients who continue to drink), evidence of chronic pancreatic disease develops as a result of progressive destruction of pancreatic tissue (i.e., parenchyma). Patients seek medical attention for persistent pain (which often leads to narcotic addiction from excessive use of pain medication), weight loss, diabetes, and maldigestion of food (a result of inadequate production of digestive enzymes by the pancreas). Abstinence from alcohol has been shown to slow the rate of progression of the disease and decrease the severity of abdominal pain.

**Figure 2:**
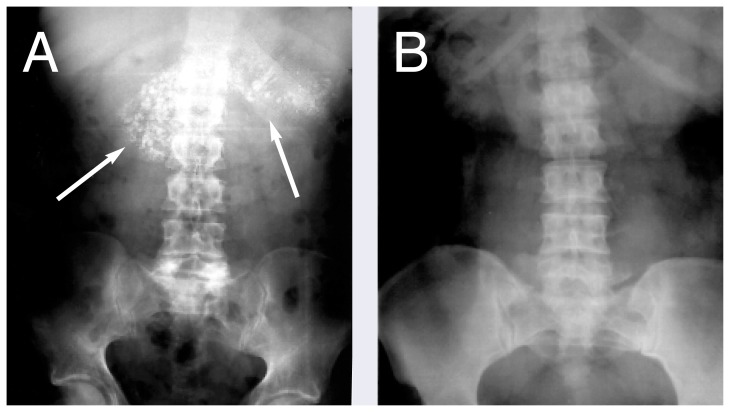
(A) Abdominal X ray of a patient with alcoholic pancreatitis. Note the speckled calcification (i.e., calcium deposits) within the pancreas (marked by arrows). (B) Abdominal X ray from a subject without pancreatitis.

Until recently, it was generally accepted that alcoholic pancreatitis began as a chronic disease with occasional episodes, or acute “flareups.” This notion was based on results of tissue analyses and x-ray studies taken from alcoholics during their first attack of pancreatitis that seemed to reveal signs of already existing chronic pancreatitis. Among these signs were shrinkage of tissue (i.e., atrophy), replacement of healthy tissue by scar tissue (i.e., fibrosis), and hardening of tissue caused by calcium deposits (i.e., calcification) (figure 2). Furthermore, autopsy studies demonstrated evidence of pancreatic fibrosis in alcoholics who had no history of clinical pancreatitis.

In recent years, the view that alcoholic pancreatitis is a form of chronic pancreatitis has been challenged. Opinion is now reverting to the hypothesis first put forward in 1946 by Comfort and colleagues, who suggested that repeated attacks of acute pancreatic inflammation *resulted* in chronic pancreatitis (Comfort et al. 1946). This hypothesis is supported by both clinical and experimental studies. A large prospective study has reported that changes in the pancreas related to chronic pancreatitis were more likely to occur in alcoholics who had recurrent acute inflammation of the pancreas (Ammann and Muellhaupt 1994). In addition, a post mortem study of 247 patients with fatal alcoholic pancreatitis demonstrated that in 53 percent of the patients, no evidence existed of chronic changes in the pancreas. Experiments show that repeated episodes of acute pancreatitis in rats produce chronic changes in the pancreas, including fat deposits, atrophy, and fibrosis (Elsasser et al. 1992).

## Diagnosis of Alcoholic Pancreatitis

A clinical diagnosis of pancreatitis is usually made on the basis of an attack of severe abdominal pain and tenderness, accompanied by a rise in the blood level of a pancreatic enzyme that digests starch (i.e., amylase) to more than three times the normal limit. Increased amylase in the blood has been the “gold-standard” diagnostic test for acute pancreatitis for more than 50 years. However, recent studies indicate that up to one-third of patients with alcoholic pancreatitis may fail to show any significant rise in amylase levels. In such circumstances, measurement of blood levels of a pancreatic enzyme that digests fats (i.e., lipase) can be helpful, because serum lipase levels remain elevated for a longer period than do amylase levels.

The diagnosis of pancreatitis may be confirmed using imaging techniques, such as X ray of the pancreas (which may reveal calcification); ultrasound examination (which provides a two-dimensional image of the pancreas); and computed tomography (CT) to detect calcification and pseudocysts. The latest development in imaging techniques for pancreatic disorders is magnetic resonance cholangiopancreatography (MRCP). This technique involves subjecting the body to a magnetic field and radio-frequency signals and provides excellent cross-sectional images of the pancreas and its main duct. It has the potential to become the diagnostic technique of choice for patients with suspected pancreatic disorders.

All the investigations described above provide structural, but not functional, information about the pancreas. To determine pancreatic function, other investigations—whether invasive or noninvasive—are necessary. Invasive methods involve stimulating the pancreas to secrete its enzymes and other fluids, collecting the fluids, and analyzing them for enzyme and bicarbonate concentration. Pancreatic insufficiency is indicated by a low secretion rate of pancreatic fluids and/or decreased enzyme and bicarbonate levels in the fluids.

One noninvasive method for testing pancreatic function is by testing for the presence of steatorrhoea. Still another, more recent, method involves oral administration of a substance that requires pancreatic enzymes for its breakdown. The amount of the breakdown product subsequently detected in breath or urine is compared with values found in people with normal pancreatic function. The main disadvantage of functional tests, however, is that they often yield false-normal results in cases with mild pancreatic dysfunction and false-abnormal results in the presence of diseases of other organs, such as the liver, lungs, and kidneys.

The cause of a case of pancreatitis can be attributed to alcohol based on a patient’s history of alcohol abuse. Attempts are under way to find a biochemical marker that would help distinguish alcoholic from nonalcoholic pancreatitis. One report has suggested that the ratio of serum lipase to serum amylase levels may be helpful in this regard (Gumaste et al. 1991). Subsequent investigations, however, have found that this ratio is not sufficiently sensitive or specific for determining the cause of pancreatitis (King et al. 1995).

Another potentially useful biochemical test has been described recently. A Belgian study demonstrated that elevated activity of trypsin, a pancreatic enzyme that digests protein, is specifically associated with acute alcoholic pancreatitis (Le Moine et al. 1994). These researchers found that activity of trypsin in the blood increased in every study subject with alcoholic pancreatitis, even when the amylase and lipase levels were normal. Conversely, serum trypsin activity did not differ between healthy controls, alcoholic controls, and patients with nonalcoholic pancreatitis. However, this was a small study, with only 32 patients in the experimental group. Larger studies are needed to confirm the usefulness of serum trypsin as a specific marker of alcohol-related pancreatic disease.

## Treatment of Alcoholic Pancreatitis

The mainstays of treatment for an acute attack of alcoholic pancreatitis are bed rest, pain relief, fasting, and administration of intravenous fluids. Other treatment measures, such as the administration of enzyme inhibitors (to reduce the corrosive effects of digestive enzymes on the pancreas) and the administration of chemicals that protect against dangerously reactive molecular fragments (i.e., antioxidants) are not yet of proven benefit. Similarly, it is not yet known whether protective (i.e., prophylactic) antibiotics have any place in the routine treatment of acute pancreatitis. Two controlled trials of prophylactic antibiotic treatment in severe pancreatitis have demonstrated a significant reduction in secondary systemic infection (i.e., septic episodes), although the treatment did not alter the death rate or the need for surgery in these patients (Pederzoli et al. 1993; Delcenserie et al. 1996). Surgery is required to manage complications such as pseudocysts and pancreatic abscesses and is sometimes needed for the treatment of chronic pain.

The treatment of chronic alcoholic pancreatitis is difficult. Abstinence from alcohol reduces the frequency of acute attacks as well as decreases pain. The pain of chronic pancreatitis can be controlled by medication (preferably nonnarcotics). The clinician first must rule out other possible causes of pain in these patients, such as pseudocysts, tumors, or ulcers. In some cases, intractable pain can be temporarily relieved by chemically blocking the nerves that supply sensation to the pancreas. Poor pancreatic function (e.g., impaired enzyme excretion) is often treated by administering pancreatic enzyme preparations in tablets or capsules, whereas diabetes is treated with oral hypoglycemic agents or insulin.

## How Alcoholic Pancreatitis May Develop

Despite decades of research, the pathogenesis of alcoholic pancreatitis remains elusive. Studies have been hampered because little is known about the earliest effects of alcohol on the human pancreas and because obtaining human pancreatic tissue for examination during life is difficult, because of its relatively inaccessible position within the abdomen. The slow progress in this field also can be attributed to the lack of a suitable animal model. Nonetheless, significant advances have been made, particularly with respect to the direct toxic effects of alcohol on acinar cells.

Early theories regarding the development of alcoholic pancreatitis focused on the main pancreatic duct, which carries pancreatic juices to the small intestine, and a muscular structure where the pancreatic duct opens into the small intestine (i.e., the sphincter of Oddi). In the 1970’s, the research emphasis shifted to the small ducts that lead to the main pancreatic duct. In recent years, however, the focus has changed again, with most research centering on the alcohol’s direct effects on acinar cells. These theories are discussed below.

### Alcohol and the Large Pancreatic Duct

One early theory postulated that pancreatic injury is caused by alcohol-induced spasm of the sphincter of Oddi, leading to backup of pancreatic enzymes into the unprotected tissues of the pancreas. Therefore, instead of entering the intestine to digest food, the enzymes “digest” the pancreatic cells themselves. Another theory postulated that backflow of bile or the contents of the duodenum into the pancreatic duct led to pancreatic damage. However, studies to date have failed to provide convincing data to support these theories.

### Effects of Alcohol on Small Ducts

Small pancreatic ducts begin at the acini and drain into the large pancreatic duct. In the early 1970’s researchers hypothesized that alcohol induces pancreatitis by causing small pancreatic ducts to be blocked by protein plugs. According to this hypothesis, the acini that secrete into the blocked ducts would then undergo fibrosis, while the plugs would eventually enlarge and calcify. Research has not clearly demonstrated that protein deposition within pancreatic ducts precedes acinar damage. It is therefore uncertain whether protein plugs are a cause or an effect of pancreatic injury. Nonetheless, it is generally accepted that protein plugs may play an important role in the progression, if not the initiation, of the disease.

Protein plugs are composed of pancreatic digestive enzymes and two other pancreatic secretory proteins, lithostathine and GP2 (Freedman et al. 1993). These two proteins possess unique properties that may be important to the process of protein-plug formation.

#### Pancreatic Lithostathine

Lithostathine is a proteinlike substance that forms 5 to 10 percent of the protein in pancreatic secretions. Lithostathine has two properties that make it relevant to the protein plug theory. First, lithostathine inhibits the deposition of calcium from pancreatic juice (Bernard et al. 1992). Therefore, a decrease in the level of lithostathine could promote calcification of protein plugs. Second, enzymes may convert lithostathine to lithostathine S1, which forms deposits spontaneously in pancreatic juice, possibly forming a starting point for further protein plug formation.

Reports conflict on the levels of lithostathine in the pancreatic fluids or tissue of patients with alcoholic pancreatitis. A recent study (Apte et al. 1996) has shown that long-term alcohol administration significantly increases one of the factors that regulate lithostathine synthesis (i.e., lithostathine mRNA levels). The consequent increase in synthesis of lithostathine could, in turn, lead to increased concentrations of lithostathine in pancreatic juice. The action of enzymes on lithostathine in the juice may promote protein deposition in ducts.

#### Pancreatic GP2

The protein GP2 is another protein consistently found in protein plugs and in calcifications from the pancreatic ducts of patients with alcoholic pancreatitis. When the pancreas is stimulated (as by consuming a meal), GP2 is discharged, along with digestive enzymes from the acinar cells. GP2 tends to aggregate in pancreatic juice (Freedman et al. 1993) and may encourage further protein precipitation. An increase in GP2 concentration in pancreatic juice would therefore favor protein plug formation.

### Direct Toxic Effects of Alcohol on Acinar Cells

Most recent research into the pathogenesis of alcoholic pancreatitis has centered on the direct toxic effects of alcohol on acinar cells. This direction of research is not unreasonable given that the acinar cell synthesizes large amounts of digestive enzymes, which have the potential to cause cell injury when activated (see below).

#### Role of Digestive Enzymes in Pancreatic Injury

A single pancreatic acinar cell can synthesize and secrete up to 10 million enzyme molecules per day. The acinar cell is normally protected from digesting itself by synthesizing most digestive enzymes as inactive precursors (i.e., zymogens), by segregating zymogens within membranous compartments (i.e., zymogen granules), and by producing protective enzymes that destroy digestive enzymes. Any disruption of these normal protective mechanisms could result in premature activation of zymogens and subsequent “autodigestive” injury. Substantial evidence supports a role for active digestive enzymes, such as trypsin, in pancreatic injury. Perhaps the most compelling evidence that active trypsin plays a role in pancreatitis is the recent discovery of a mutant gene in patients with hereditary pancreatitis (Whitcomb et al. 1996). This mutation produces a trypsin variant that cannot be degraded by the acinar cell’s protective enzymes. The consequent accumulation of active trypsin could initiate activation of other enzymes, resulting in the autodigestion of the pancreas.

#### Effect of Alcohol on Pancreatic Enzymes

Long-term alcohol consumption may lead to premature activation of digestive enzymes in the acinar cell. In this regard, it has been shown that alcohol increases the synthesis of digestive enzymes in the pancreas (Wilson et al. 1992; Apte et al. 1995) and increases the fragility of the zymogen granules (Haber et al. 1994), potentially allowing zymogens to leak into the cell. In addition, alcohol consumption increases the fragility of lysosomes, structures that, like zymogen granules, sequester lysosomal enzymes within the cell. The lysosomal enzyme cathepsin B is capable of activating the digestive enzyme trypsinogen to its active form, trypsin.

The increase in lysosomal fragility appears to be mediated by two compounds known to accumulate in the pancreas after chronic alcohol consumption: cholesteryl esters and fatty acid ethyl esters (FAEE’s) (Wilson et al. 1992; Haber et al. 1993). The mechanism responsible for the alcohol-induced increase in zymogen granule fragility is not yet understood. One possibility is an alcohol-induced reduction in GP2 content of zymogen granule membranes (as has been demonstrated recently in alcohol-fed rats [Apte et al. in press*b*]). As noted earlier, GP2 is a major component protein of the zymogen granule membrane and is postulated to play an important role in zymogen granule maturation and stability.

#### Alcohol-Induced Oxidant Stress

Reactive oxygen species, or free radicals, are unstable molecules that are generated as by-products of normal metabolic processes. These molecules may damage cell membranes, proteins, and genetic material through the process of oxidation. The cell is normally protected from the disruptive effects of free radicals by chemical antioxidant systems. An imbalance between free-radical production and the antioxidant capability of a cell leads to oxidant stress within the cell.

Oxidant stress has been implicated as a possible mechanism of pancreatitis. Metabolism of alcohol by an enzyme in the liver called cytochrome P450 2E1 leads to the generation of free radicals. Normally functioning at a low level, this enzyme system can be activated (i.e., induced) by heavy alcohol consumption and is a major pathway for alcohol metabolism in the liver in heavy drinkers (Lieber 1992). Recent evidence demonstrates that cytochrome P450 2E1 also is present in the pancreas and, moreover, is induced by chronic alcohol administration (Norton et al. 1996). In addition, acute alcohol administration increases levels of compounds formed by the reaction of free radicals with membrane components (i.e., lipid peroxidation products) in rat pancreas, thus providing direct evidence that alcohol causes oxidant stress within the pancreas (Altomare et al. 1996).

#### Effects of Toxic Metabolites of Alcohol

Metabolism of alcohol by the liver—with consequent production of toxic metabolites, such as acetaldehyde and FAEE’s—has been shown to play a central role in alcoholic liver disease (Lieber 1991). Acetaldehyde binds to liver proteins, altering their function and inducing a damaging immune response. FAEE’s can disrupt membranes within the cell.

Similar metabolic events may occur in the pancreas exposed to alcohol. A recent study, using cultures of rat pancreatic acinar cells, has shown that at intoxicating alcohol concentrations, acinar cells metabolize significant amounts of alcohol (Haber et al. 1995*b*). The rate of this metabolism approaches that of liver cells and can potentially contribute to pancreatic cellular injury. In addition, both human and rat pancreas can synthesize FAEE’s in the presence of alcohol (Apte et al. in press*a*).

**Figure 3:**
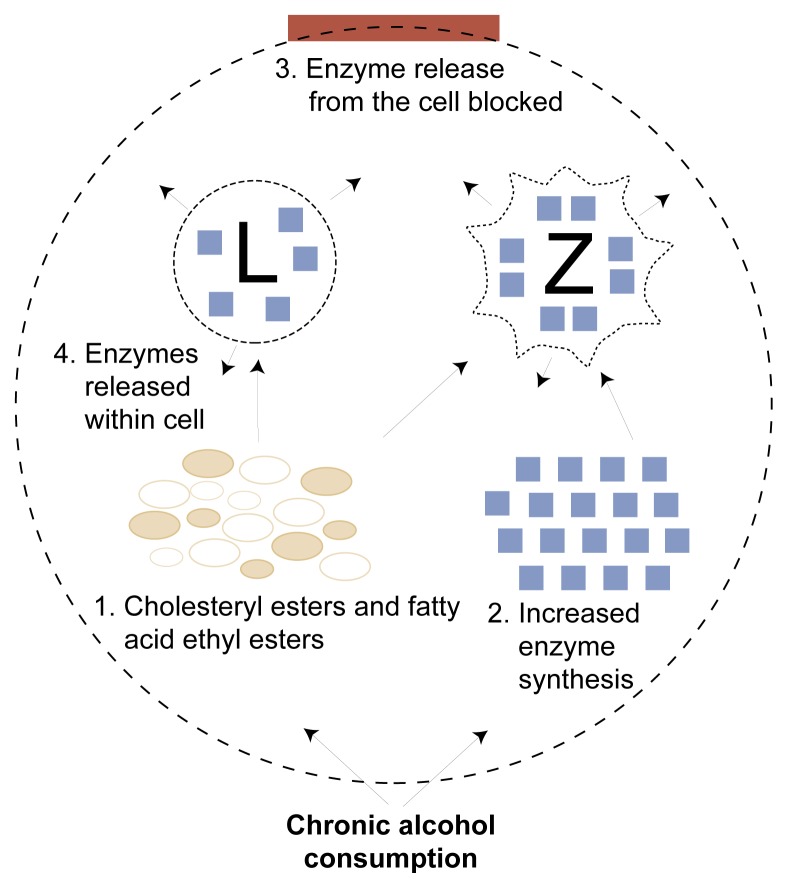
The metabolic effects of alcohol on pancreatic cells may lead to digestion of the cell. (1) Chronic alcohol consumption increases cholesteryl esters and fatty acid ethyl esters in organelle membranes, altering the fragility of enzyme storage structures within the cell (i.e., lysosomes [L] and zymogen granules [Z]). (2) Chronic alcohol consumption increases digestive enzyme synthesis. (3) Chronic alcohol consumption blocks the release of digestive enzymes from the cell. (4) Release of digestive enzymes from fragile L and Z granules into the cell’s interior breaks down cell components (i.e., autodigestion).

In summary, increasing evidence suggests that direct toxic effects of alcohol or the products of its metabolism play a major role in alcoholic pancreatitis. This knowledge has led to the concept of the “drinker’s pancreas” (figure 3) in which the effects of alcohol and its metabolic by-products (including reactive oxygen species) lead to excessive accumulation of digestive and lysosomal enzymes in the acinar cell (through increased synthesis and, possibly, decreased secretion). In addition, cholesteryl esters, FAEE’s, and reactive oxygen species increase the fragility of zymogen granules and lysosomes, thereby increasing the potential for contact between digestive and lysosomal enzymes. These changes occur in the absence of overt pancreatic damage, suggesting that an additional trigger factor may be required to initiate injury.

## Factors Influencing Individual Susceptibility

An apparent clinical paradox exists with respect to the occurrence of pancreatitis in alcoholics. Although it is well established that the risk of developing pancreatitis rises with increasing alcohol consumption (suggesting the presence of constant dose-related effects of alcohol on the pancreas), it is also clear that only a small proportion of heavy drinkers develop clinically significant pancreatitis. The latter observation raises the possibility that a factor (or factors) other than alcoholism influence the susceptibility of an alcoholic to pancreatitis. A number of factors that may distinguish alcoholics who develop pancreatitis from those who do not have been investigated (Haber et al. 1995*a*). These factors include diet, amount and type of alcohol consumed, the pattern of alcohol consumption, hereditary factors (e.g., blood group), fat intolerance, and smoking. Many studies have provided conflicting results, probably because they compared subjects with alcoholic pancreatitis (i.e., the experimental group) with subjects from the general population (i.e., the control group). Thus, the control and experimental groups differed from each other with respect to two of the factors under study: alcoholism and pancreatitis.

The essential comparison in such studies must be between alcoholics *with* the disease and alcoholics *without* the disease. Thus, the only difference between the experimental and the control groups should be the presence or absence of pancreatitis. When possible predisposing factors were studied in this controlled fashion, no consistent association could be detected between them and alcoholic pancreatitis (Haber et al. 1995*a*). Thus, the factors that may make some heavy drinkers susceptible to pancreatitis have not yet been identified. Possible predisposing factors that may be the subject of future research include those that may influence the pathways of alcohol metabolism, as well as those that may increase the likelihood of enzyme-related injury to the pancreas, such as genetically determined alterations in pancreatic digestive enzymes and their inhibitors.

## Conclusion

Although the mechanism(s) responsible for the development of pancreatitis in alcoholics need(s) to be fully clarified, significant progress in this direction has been made in the past decade, particularly with respect to understanding the direct toxic effects of alcohol on the pancreas. These effects may create a “primed” setting within the pancreas, which, in the presence of an additional (as yet unidentified) trigger factor, could lead to acute, clinically evident pancreatic injury. Repeated episodes of acute pancreatic injury may lead to chronic disease. Progression of alcoholic pancreatitis may also be aided by alcohol-induced deposition of protein plugs within small pancreatic ducts. Thus, the various theories of the development of alcoholic pancreatitis need not be mutually exclusive. Indeed, it is likely that a combination of the postulated mechanisms described in this article is responsible for the manifestations of alcoholic pancreatitis.

## Footnotes

1 A standard drink is roughly equivalent to the amount of alcohol contained in 12 ounces of beer, 5 ounces of wine, or one and a half ounces of 80-proof distilled spirits.
